# Supplementary material for: Nucleosome dynamics render heterochromatin accessible in living human cells
Source: Nat Commun. 2025 May 16;16:4577. doi: 10.1038/s41467-025-59994-7 (PMC12084565; doi:10.1038/s41467-025-59994-7)
Supplement: Supplementary file 1 — Supplementary Information [file 41467_2025_59994_MOESM1_ESM.pdf]

SUPPLEMENTARY INFORMATION

Nucleosome dynamics render heterochromatin accessible in living human cells

Hemant K. Prajapati, Zhuwei Xu, Peter R. Eriksson and David J. Clark

Division of Developmental Biology, *Eunice Kennedy-Shriver* National Institute of Child Health and Human Development, National Institutes of Health, Bethesda MD 20892, USA.

Supplementary Figures 1 – 10

Supplementary References

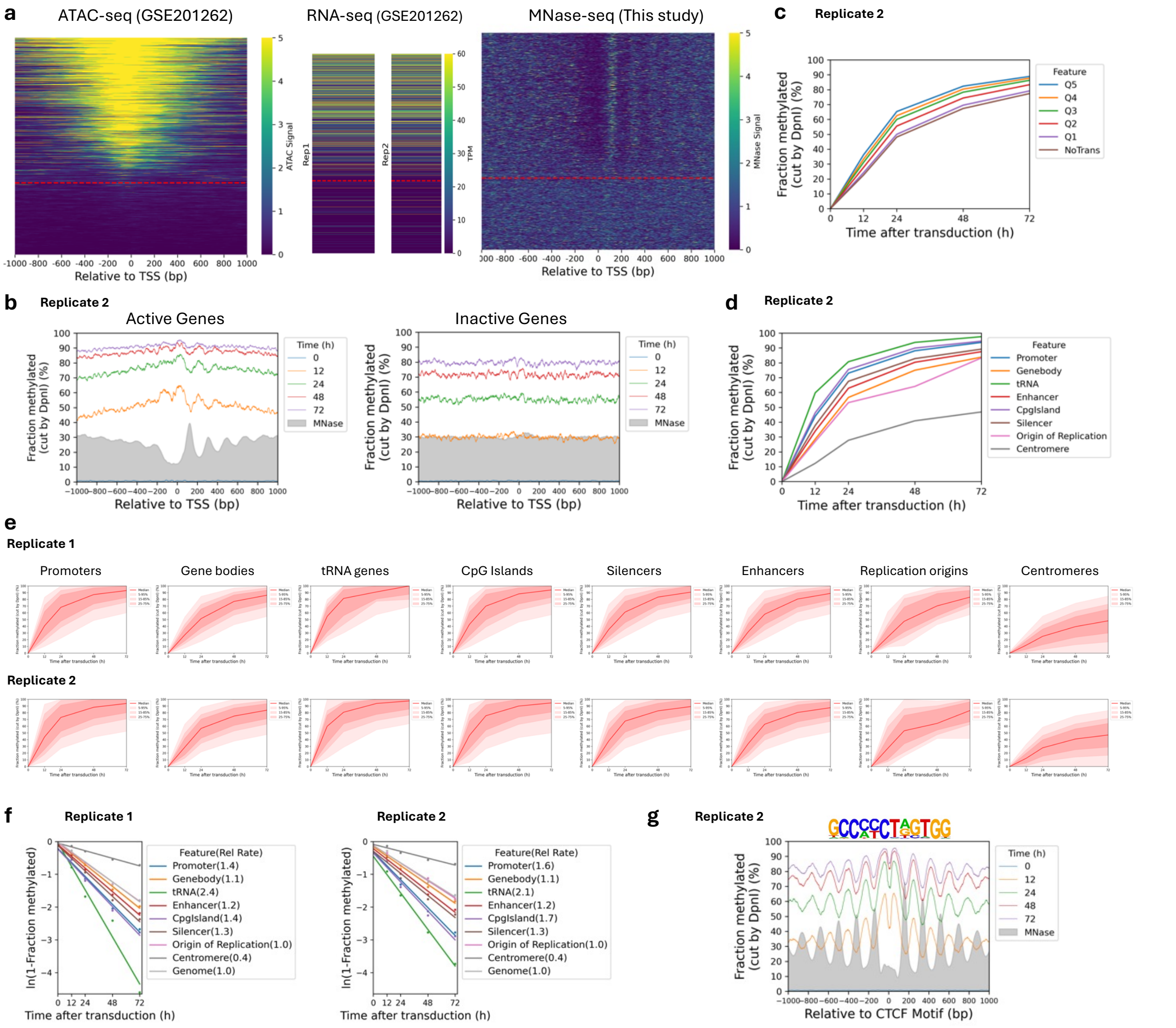

Supplementary Figure 1 | Rates of Dam methylation in various genomic regions in MCF7 cells. **a,** Left panel: All human genes sorted by ATAC-seq signal at their promoters in MCF7 cells [1] relative to the major TSS. The red line separates genes with NDRs (active) from those that have no NDR (inactive). Middle panel: RNA-seq data for MCF7 cells (from [1]) sorted as in the left panel. Right panel: MNase-seq data sorted as in the left panel. **b,** Nucleosome phasing in vivo detected by Dam methylation for Replicate 2 (see Fig.1e,f for Replicate 1). Methylation data for GATC sites across active and inactive genes at each time point are plotted relative to the TSS (smoothed with a 21-bp window). Grey profile: nucleosome dyad distribution in nuclei (MNase-seq data normalised to 30%). **c,** Effect of transcription on median GATC site methylation. The active genes were divided into quintiles, Q1 to Q5, with increasing transcriptional activity; inactive genes were treated as a single group ("NoTrans"). Data for Replicate 2 (see Fig.1g for Replicate 1). **d,** Methylation time courses for the median GATC site in various genomic regions. Data for Replicate 2 (see Fig.1h for Replicate 1). **e,** Methylation time courses for various genomic regions defined by hg38 annotations. Red line: median GATC site; shading: data range as indicated. **f,** Relative methylation rates for various genomic regions in vivo. Rates are relative to the genomic average for all GATC sites. **g,** Nucleosome phasing around CTCF motifs in vivo using the motif shown. Data for Replicate 2 (see Fig.1i for Replicate 1).

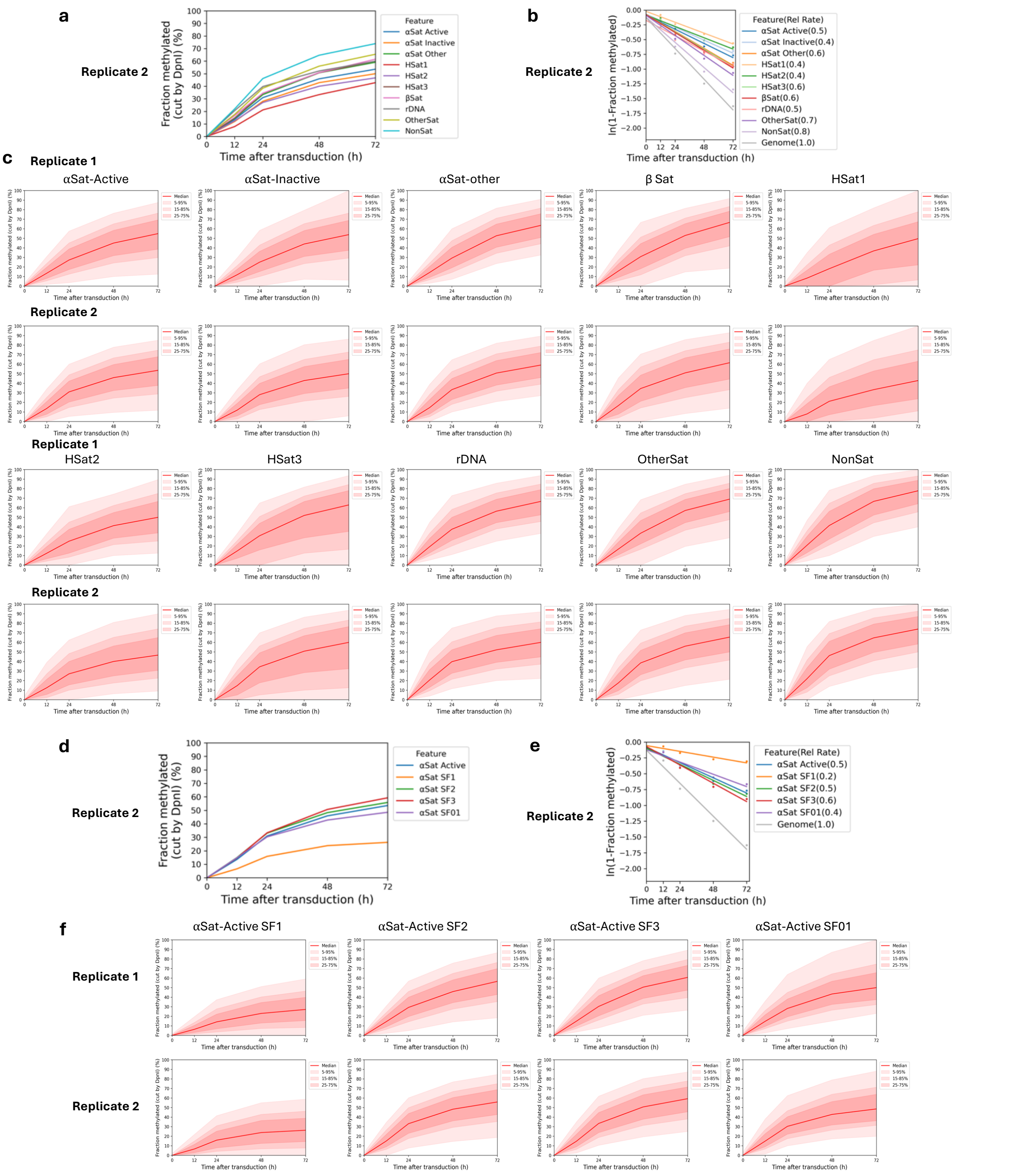

Supplementary Figure 2 | Dam methylation of the various centromeric satellite repeats in MCF7 cells. a, Methylation time courses for the median GATC site and **b,** methylation rates for the various centromeric elements relative to the genomic average site. Data for Replicate 2 (see Fig. 2c,d for Replicate 1). **c,** Methylation time courses for the median GATC site for the various centromeric elements. Red line: median GATC site; shading: data range as indicated. **d,** Methylation time courses for the median GATC site and **e,** relative methylation rates for the various active α-satellite supra-chromosomal families. Data for Replicate 2 (see Fig. 2e,f for Replicate 1). **f,** Methylation time courses for the median GATC site for the various active α-satellite supra-chromosomal families. Red line: median GATC site; shading: data range as indicated.

Feature	Total Size (bp)	Total GATC sites	GATC density (per kb)	After filtering No. of GATC sites	After filtering % GATC sites	After filtering GATC site density (per kb)	After filtering Relative Rate Rep1	After filtering Relative Rate Rep2
Genome	3088269832	7199434	2.3	5777618	80	1.9	1.0	1.0
Gene bodies	1045559149	2664781	2.5	2120994	80	2.0	1.1	1.1
Gene bodies Active (ATAC)	733627301	1894051	2.6	1496969	79	2.0	1.1	1.2
Gene bodes Inactive (ATAC)	311931848	770730	2.5	624025	81	2.0	1.0	1.0
Gene bodies NoTrans (RNA)	153677376	377462	2.5	306383	81	2.0	0.9	0.9
Gene bodies RNA Q1	225746805	553817	2.5	449536	81	2.0	1.0	1.0
Gene bodies RNA Q2	197526137	495893	2.5	397691	80	2.0	1.1	1.1
Gene bodies RNA Q3	198298996	512088	2.6	404960	79	2.0	1.2	1.2
Gene bodies RNA Q4	171069359	452517	2.6	353479	78	2.1	1.2	1.3
Gene bodies RNA Q5	99240476	273004	2.8	208945	77	2.1	1.3	1.3
Promoters	4056200	6995	1.7	4924	70	1.2	1.4	1.6
Promoters Active (ATAC)	2713400	4313	1.6	2785	65	1.0	2.4	2.7
Promoters Inactive (ATAC)	1342800	2682	2.0	2139	80	1.6	1.1	1.1
Promoters NoTrans (RNA)	753800	1523	2.0	1240	81	1.6	1.0	1.1
Promoters RNA Q1	660600	1195	1.8	882	74	1.3	1.2	1.4
Promoters RNA Q2	660400	1095	1.7	785	72	1.2	1.5	1.7
Promoters RNA Q3	660400	1094	1.7	696	64	1.1	1.7	2.7
Promoters RNA Q4	660400	1101	1.7	688	62	1.0	2.4	2.8
Promoters RNA Q5	660600	987	1.5	633	64	1.0	2.4	2.7
CpG Islands	21818820	40163	1.8	21917	55	1.0	1.5	1.7
Enhancers	51077692	115208	2.3	90494	79	1.8	1.2	1.2
Origins of replication	60608	153	2.5	123	80	2.0	1.0	1.0
Silencers	1400893	3046	2.2	2483	82	1.8	1.3	1.3
tRNA genes	32259	170	5.3	82	48	2.5	2.4	2.1
Centromeres	62154366	45277	0.7	31479	70	0.5	0.3	0.4
SF01 repeats	4214705	2419	0.6	2403	99	0.6	0.3	0.4
SF1 repeats	20802862	24048	1.2	3738	16	0.2	0.2	0.2
SF2 repeats	26541698	13000	0.5	9559	74	0.4	0.4	0.5
SF3 repeats	10053488	11830	1.2	11578	98	1.2	0.5	0.6

Supplementary Figure 3 | GATC site densities of various genomic regions. Site counts are for the hg38 genome, except for the SF repeats, which are for the T2T genome. Site densities are quoted as the number of GATC sites per kb. Filtering refers to the exclusion of GATC sites which overlap with CpG sites (excluded because they cannot be cut by DpnI if methylated (m^5CpG)), or if they are too close to neighbouring GATC sites on both sides (< 150 bp). Methylation rates relative to the genome average (1.0) are also given for MCF7 cells (replicates 1 and 2).

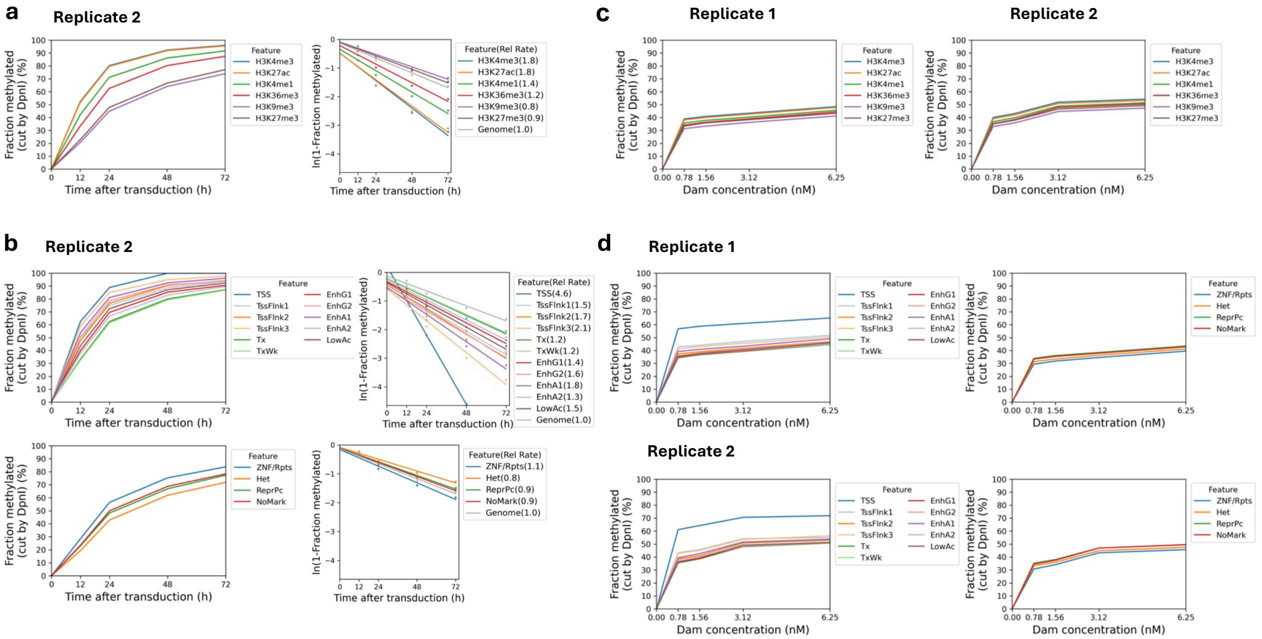

Supplementary Figure 4 | Dam methylation of heterochromatin and euchromatin in living MCF7 cells and in MCF7 nuclei. a, Methylation time courses for the median GATC site and methylation rates for regions marked by histone modifications associated with euchromatin (H3K4me1, H3K4me3, H3K27ac or H3K36me3) or heterochromatin (H3K9me3 or H3K27me3), relative to the genomic average site. ChIP-seq data from [2]. Data for Replicate 2 (see Fig. 3a,b for Replicate 1). **b,** Methylation time courses for the median GATC site and relative methylation rates for the 15 epigenetic states defined by our ChromHMM model (see Fig. 3b). Data for Replicate 2 (see Fig. 3c,d for Replicate 1). **c,** Methylation of the median GATC site in nuclei for regions marked by histone modifications associated with euchromatin or heterochromatin. **d,** Dam methylation in isolated nuclei of the different chromatin states specified by the ChromHMM model (see Fig. 3b).

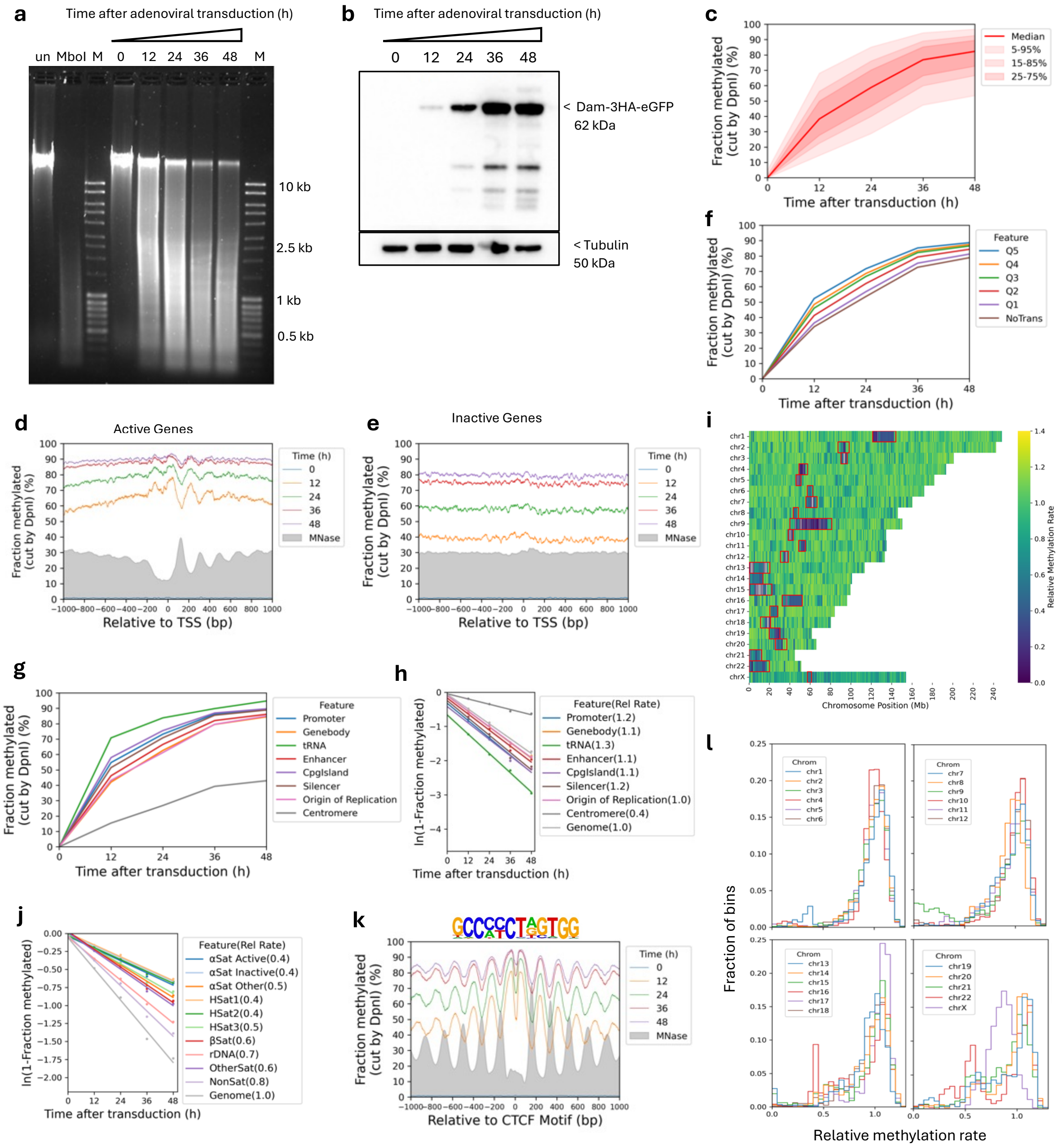

Supplementary Figure 5 | The human genome is globally accessible in live MCF10A cells. a, Agarose gel electrophoresis of DpnI-digested genomic DNA purified from MCF10A cells as a function of time of adenovirus treatment. 'un', undigested genomic DNA; 'MboI', DNA from non-transduced cells digested with MboI; M, DNA size marker. **b,** Anti-HA immunoblot to detect Dam-3HA-eGFP expression in MCF10A cells. **c,** Almost complete methylation of GATC sites in MCF10A cells after transduction. Red line and shading: median GATC site methylation with data range indicated. **d,e,** Nucleosome phasing with respect to the TSS for active and inactive genes, as defined by ATAC-seq data for MCF10A cells [3]. Grey profile: nucleosome dyad distribution in nuclei (MNase-seq data for MCF7 cells arbitrarily normalised to 30%). **f,** The effect of transcriptional activity on methylation rate. Active genes were divided into quintiles Q1 to Q5 based on increasing transcriptional activity (Q5 is the highest) using RNA-seq data for MCF10A cells from [4]; methylation of the median GATC site in each quintile is shown. Inactive genes are treated as a single separate group ('NoTrans'). **g,** Median GATC methylation for various genomic regions using annotations from the hg38 genome. **h,** Relative median GATC site methylation rates for various genomic regions. **i,** Heat map showing the variation in methylation rate at the chromosomal level in MCF10A cells. The average methylation rate was calculated for all GATC sites in each 100 kb window in the T2T genome by plotting 'ln (1- fraction methylated)' against time after adenovirus transduction, and then normalised to the genomic average rate to obtain relative rates. Red rectangles: centromeric regions. **j,** Relative methylation rates for the various centromeric elements. **k,** Nucleosome phasing around CTCF motifs in MCF10A cells using the motif shown. **l,** Relative methylation rate data derived as in 'i' for each of the 23 chromosomes are separated into four separate plots for ease of comparison. Histograms of the fraction of 100-kb windows having a given relative methylation rate.

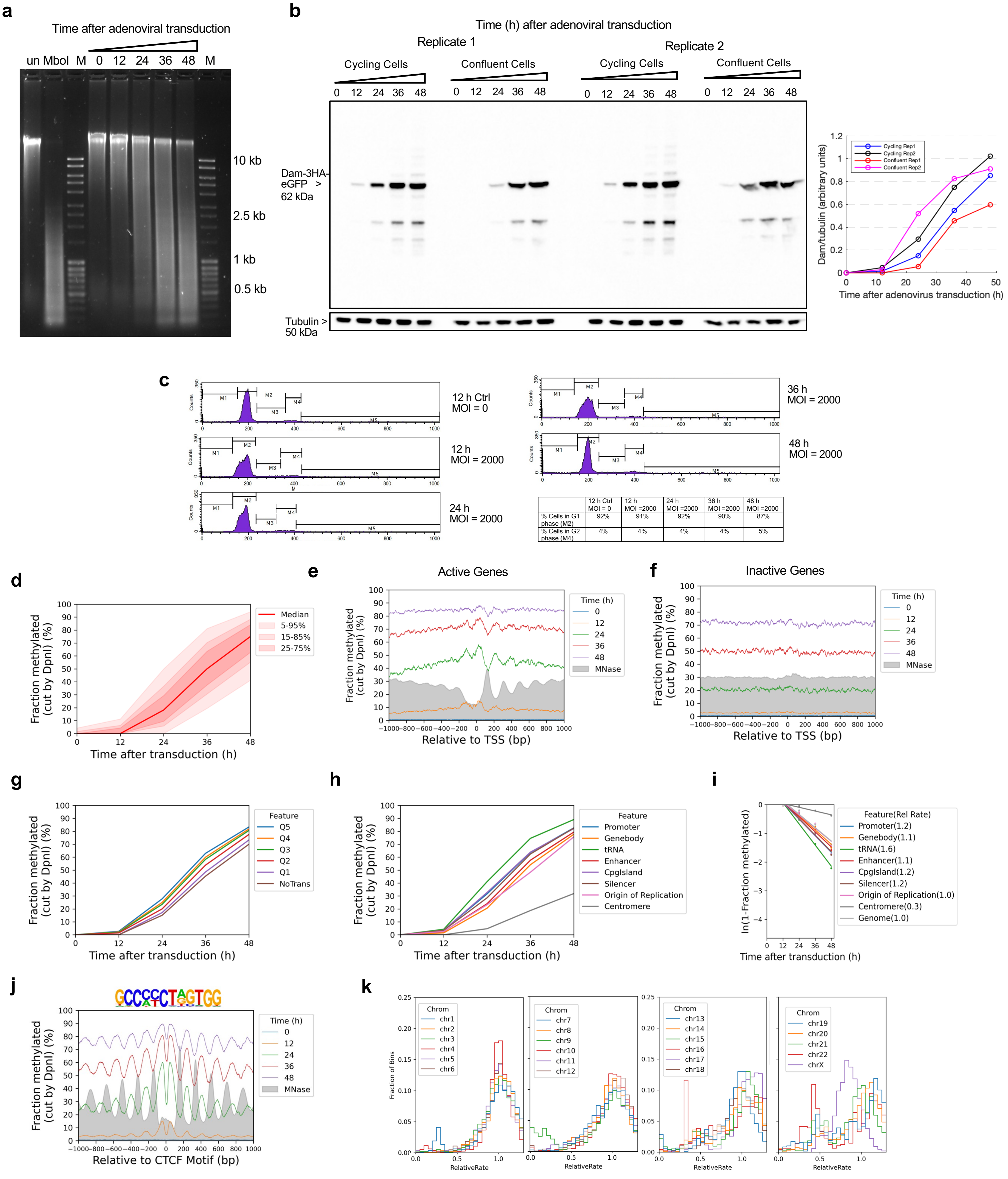

Supplementary Figure 6 (see legend on next page)

Supplementary Figure 6 | The human genome is globally accessible in confluent MCF10A cells.
a, Agarose gel electrophoresis of DpnI-digested genomic DNA purified from confluent MCF10A cells as a function of time of adenovirus treatment. 'un', undigested genomic DNA; 'MboI', DNA from non-transduced cells digested with MboI; M, DNA size marker. **b,** Anti-HA immunoblot to compare Dam-3HA-eGFP expression in cycling and confluent MCF10A cells. All samples were analysed in the same blot and quantified relative to tubulin. **c,** FACS analysis confirms that the confluent MCF10A cells are arrested in G1 during the time course after adenovirus transduction. **d,** Time course of Dam methylation of all GATC sites in MCF10A cells after transduction. Red line and shading: median GATC site methylation with data range indicated. **e,f,** Nucleosome phasing with respect to the TSS for active and inactive genes, as defined by ATAC-seq data for MCF10A cells 3. Grey profile: nucleosome dyad distribution in nuclei (MNase-seq data for MCF7 cells arbitrarily normalised to 30%). **g,** The effect of transcriptional activity on methylation rate. Active genes were divided into quintiles Q1 to Q5 based on increasing transcriptional activity (Q5 is the highest) using RNA-seq data for MCF10A cells 4; methylation of the median GATC site in each quintile is shown. Inactive genes are treated as a single separate group ('NoTrans'). **h,** Median GATC methylation for various genomic regions using hg38 genome annotations. **i,** Relative median GATC site methylation rates for various genomic regions. **j,** Nucleosome phasing around CTCF motifs in MCF10A cells using the motif shown. **k,** Histograms of the fraction of 100-kb windows having a given relative methylation rate for each of the 23 chromosomes are separated into four separate plots for ease of comparison.

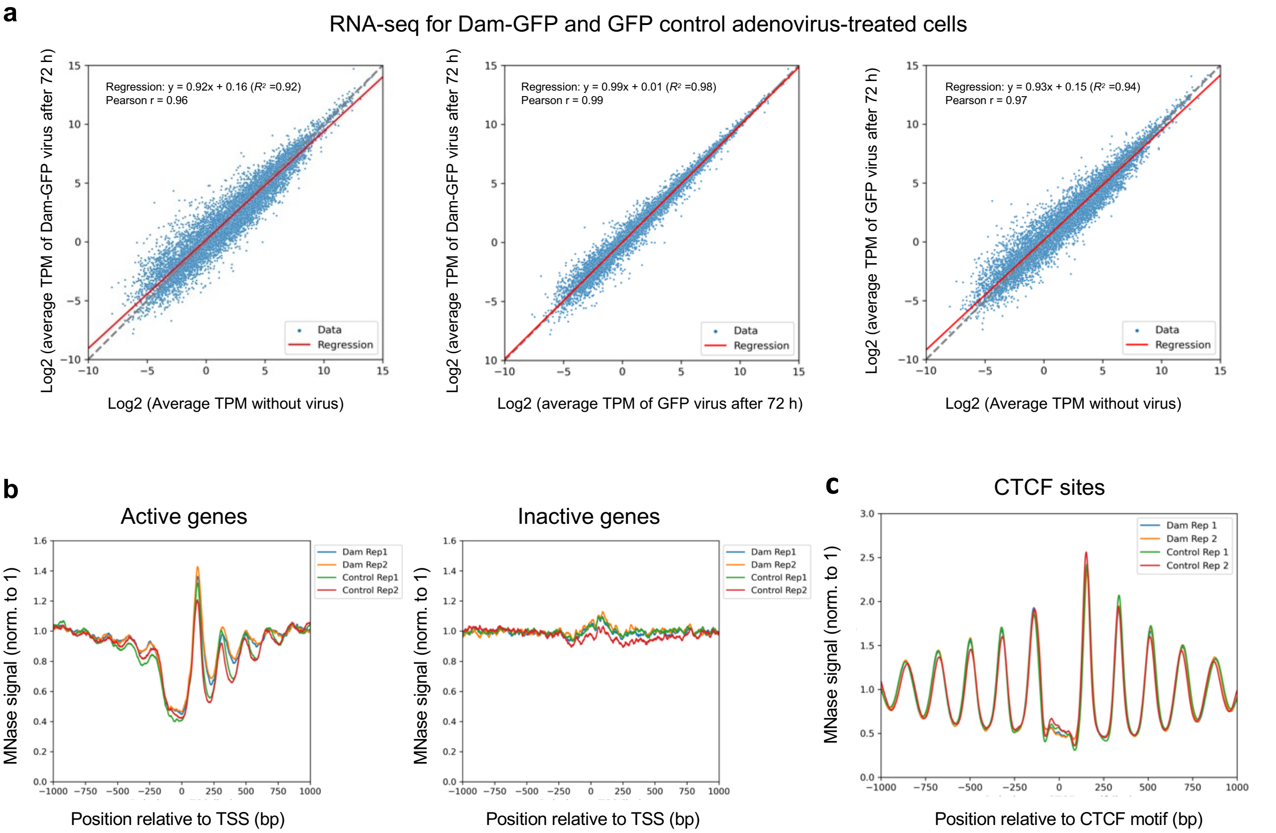

Supplementary Figure 7 | Dam-GFP adenovirus treatment has little effect on gene expression and chromatin organisation in MCF7 cells. a, Gene expression in adenovirus-treated cells. Scatter plots showing the average RNA-seq signal (from three replicate experiments) for all genes with TPM > 0 in MCF7 cells before (0 h) and after treatment (72 h), with either the Dam-GFP fusion expression adenovirus or a control GFP expression adenovirus. **b,** Chromatin organisation on active and inactive genes in MCF7 cells: MNase-seq data. Nucleosome dyad phasing plots relative to the TSS for untreated cells and cells treated with the Dam-GFP fusion virus for 72 h. **c,** Chromatin organisation flanking CTCF binding sites in Dam-GFP adenovirus-treated MCF7 cells. MNase-seq data. Nucleosome dyad phasing plots relative to the CTCF binding motif for untreated cells and cells treated with the Dam-GFP fusion virus for 72 h.

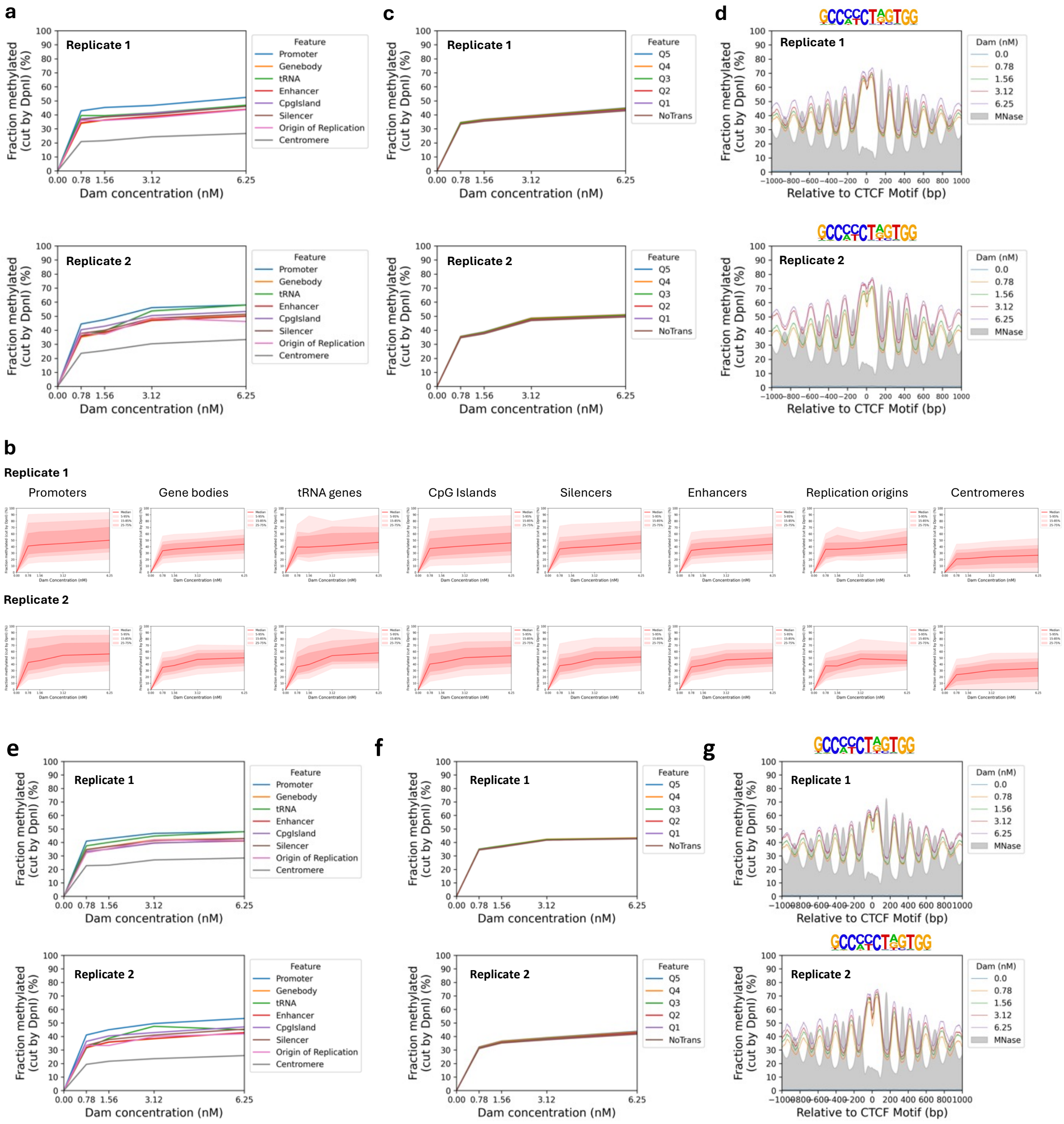

Supplementary Figure 8 | Limited genome accessibility in isolated MCF7 nuclei. Nuclei were treated with increasing concentrations of Dam with (**a-d**) or without spermidine (**e-g**) in the buffer. **a,** Comparison of the methylation of the median GATC site in various genomic regions as a function of Dam concentration. **b,** Separate plots for methylation of the median GATC site in various genomic regions as a function of Dam concentration. Red line and shading: median GATC site methylation with data range indicated. **c,** The effect of transcriptional activity on methylation rate in nuclei. Active genes were divided into quintiles Q1 to Q5 based on increasing transcriptional activity (Q5 is the highest) using RNA-seq data for MCF7 cells [1]; methylation of the median GATC site in each quintile is shown. Inactive genes are treated as a single separate group ('NoTrans'). **d,** Nucleosome phasing in nuclei relative to CTCF motifs detected by Dam methylation using the motif shown. Methylation data for GATC sites at each Dam concentration are plotted relative to each CTCF site (smoothed with a 21-bp window). Grey profile: nucleosome dyad distribution in nuclei (MNase-seq data normalised to 30%). **e,** As in 'a', but in the absence of spermidine. **f,** As in 'c', but in the absence of spermidine. **g,** As in 'd', but in the absence of spermidine.

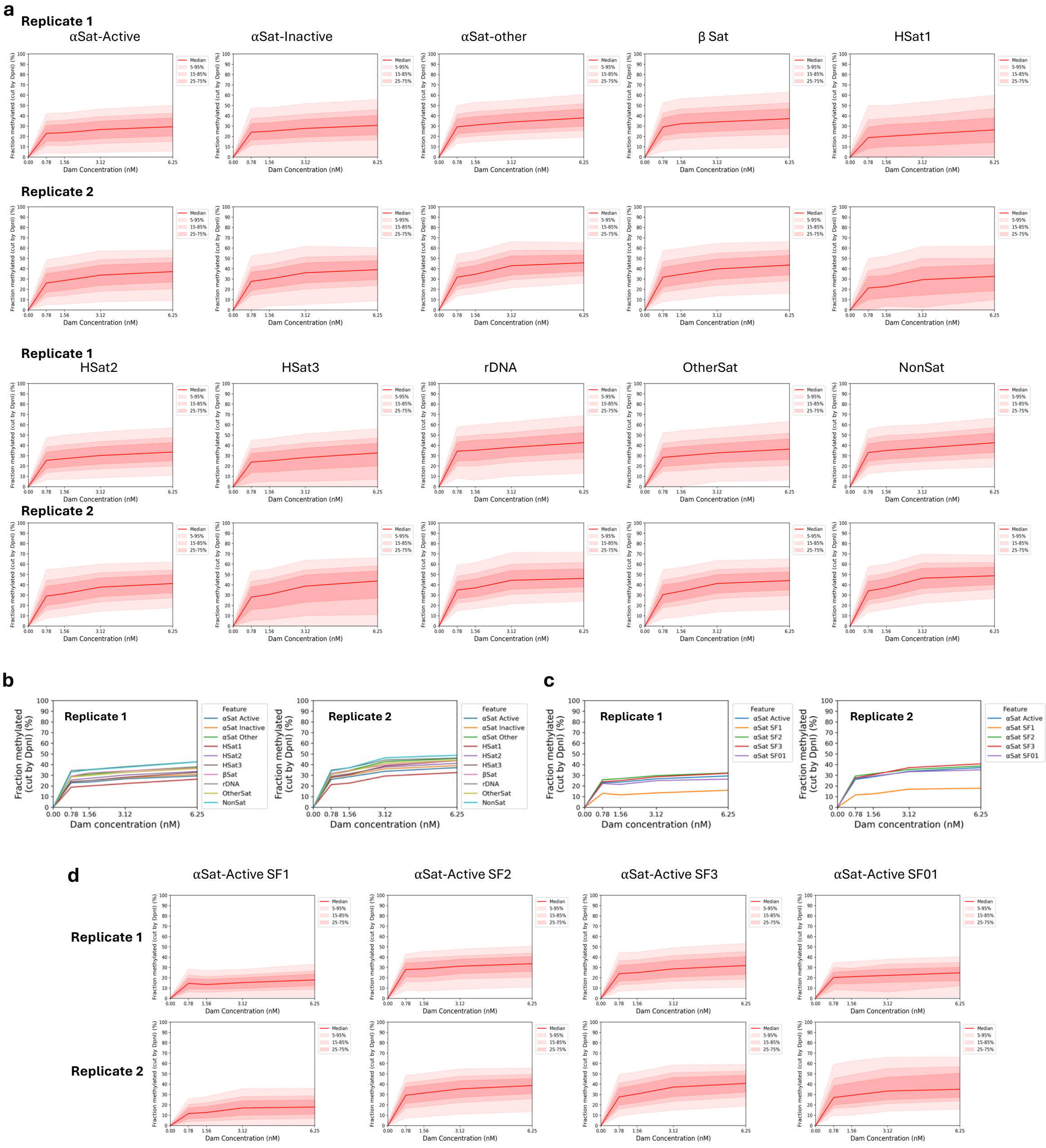

Supplementary Figure 9 | Methylation of centromeric elements is limited in nuclei. a, Separate plots showing methylation of the median GATC site in various centromeric elements as a function of Dam concentration. Red line and shading: median GATC site methylation with data range indicated. **b,** Comparison of the methylation of the median GATC site in the various centromeric elements as a function of Dam concentration. **c,** Comparison of the methylation of the median GATC site in the various active α-satellite supra-chromosomal families as a function of Dam concentration. **d,** Separate plots showing methylation of the median GATC site in various active α-satellite supra-chromosomal families as a function of Dam concentration.

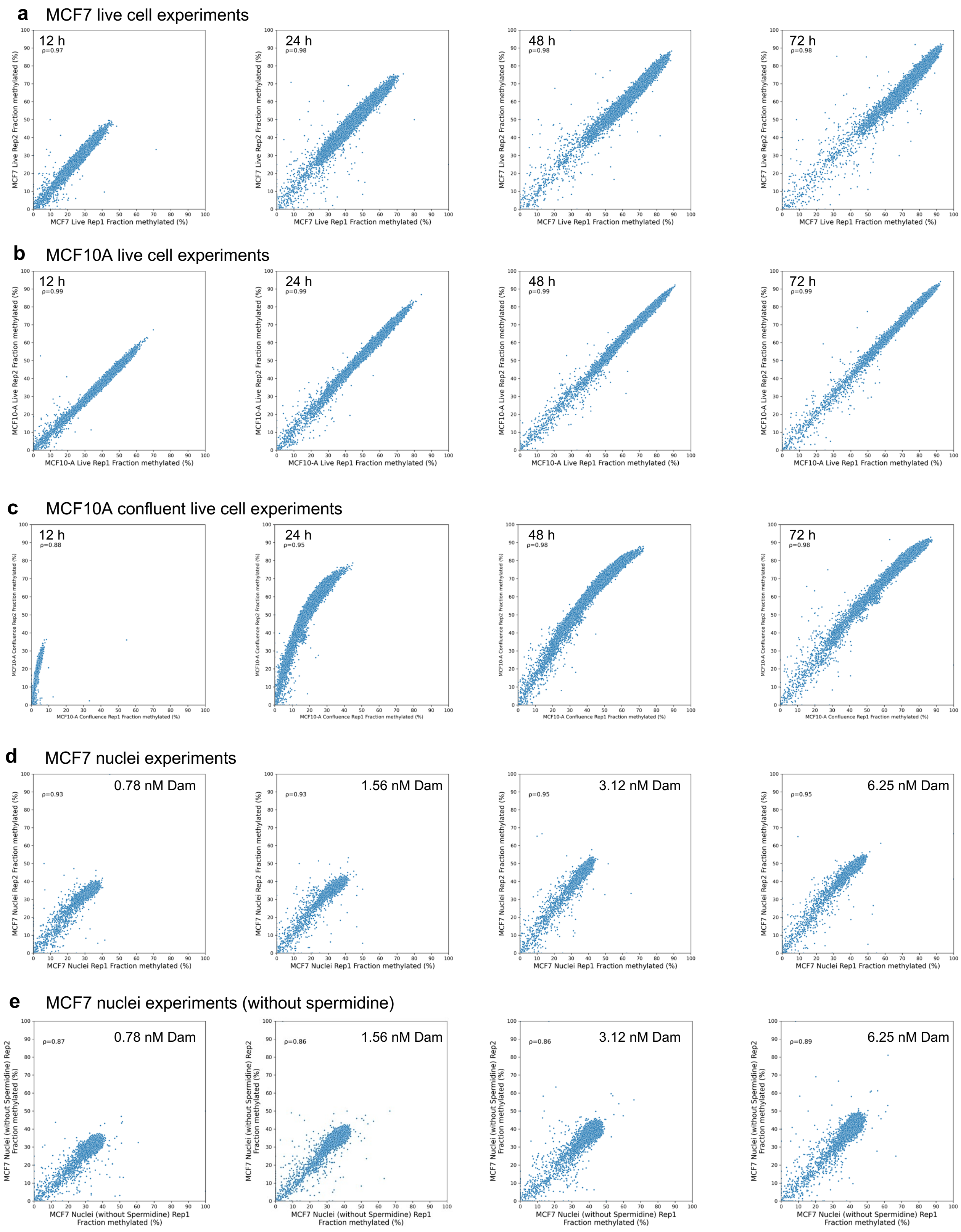

Supplementary Figure 10 | Comparison of biological replicate experiments at the chromosomal level. Pearson correlations for the average % methylated for all GATC sites in each 100 kb window for each time point (live cells) or Dam concentration (nuclei). **a,** MCF7 cells, **b,** Dividing MCF10A cells, **c,** Confluent MCF10A cells, **d,** MCF7 nuclei with spermidine in the buffer, **e,** MCF7 nuclei without spermidine in the buffer.

SUPPLEMENTARY REFERENCES

1. Tian C, *et al.* Impaired histone inheritance promotes tumor progression. *Nat Commun* **14**, 3429 (2023).

2. Franco HL, *et al.* Enhancer transcription reveals subtype-specific gene expression programs controlling breast cancer pathogenesis. *Genome Res* **28**, 159-170 (2018).

3. Gross SM, *et al.* A multi-omic analysis of MCF10A cells provides a resource for integrative assessment of ligand-mediated molecular and phenotypic responses. *Commun Biol* **5**, 1066 (2022).

4. Dorgham MG, Elliott BA, Holley CL, Mansfield KD. m6A regulates breast cancer proliferation and migration through stage-dependent changes in Epithelial to Mesenchymal Transition gene expression. *Front Oncol* **13**, 1268977 (2023).
