# Supplementary material for: Nucleosome dynamics render heterochromatin accessible in living human cells
Source: Nat Commun. 2025 May 16;16:4577. doi: 10.1038/s41467-025-59994-7 (PMC12084565; doi:10.1038/s41467-025-59994-7)
Supplement: Supplementary file 2 — Reporting Summary [file 41467_2025_59994_MOESM2_ESM.pdf]

nature portfolio

Corresponding author(s): Prajapati/ Clark

Last updated by author(s): May 1 2025

Reporting Summary

Nature Portfolio wishes to improve the reproducibility of the work that we publish. This form provides structure for consistency and transparency in reporting. For further information on Nature Portfolio policies, see our Editorial Policies and the Editorial Policy Checklist.

Statistics

For all statistical analyses, confirm that the following items are present in the figure legend, table legend, main text, or Methods section.

n/a	Confirmed	
☐	☒	The exact sample size (*n*) for each experimental group/condition, given as a discrete number and unit of measurement
☒	☐	A statement on whether measurements were taken from distinct samples or whether the same sample was measured repeatedly
☒	☐	The statistical test(s) used AND whether they are one- or two-sided<br>*Only common tests should be described solely by name; describe more complex techniques in the Methods section.*
☒	☐	A description of all covariates tested
☒	☐	A description of any assumptions or corrections, such as tests of normality and adjustment for multiple comparisons
☐	☒	A full description of the statistical parameters including central tendency (e.g. means) or other basic estimates (e.g. regression coefficient) AND variation (e.g. standard deviation) or associated estimates of uncertainty (e.g. confidence intervals)
☒	☐	For null hypothesis testing, the test statistic (e.g. *F*, *t*, *r*) with confidence intervals, effect sizes, degrees of freedom and *P* value noted<br>*Give P values as exact values whenever suitable.*
☒	☐	For Bayesian analysis, information on the choice of priors and Markov chain Monte Carlo settings
☒	☐	For hierarchical and complex designs, identification of the appropriate level for tests and full reporting of outcomes
☐	☒	Estimates of effect sizes (e.g. Cohen's *d*, Pearson's *r*), indicating how they were calculated

Our web collection on statistics for biologists contains articles on many of the points above.

Software and code

Policy information about availability of computer code

Data collection	We developed two packages for methylated fraction analysis: snakemakeMethylFrac and methylFracAnalyzer. SnakemakeMethylFrac, a snakemake workflow, processes raw Illumina paired-end reads to determine methylated fractions at all GATC sites. Bowtie2 v2.5.1 is used for alignment and bedtools v2.31.1 is used to calculate the occupancy (fragment coverage) and 5'-end counting. GATC sites that overlap with CpG sites were filtered out, because DpnI cannot cut GATm5C. GATC half-sites that are within 150 bp of each other were also filtered out because small DNA fragments < 150 bp tend to be lost during sample purification. The output includes SQLite database and bigwig files. We use pandas, pyBigWig (https://github.com/deeptools/pyBigWig), biopython, matplotlib and seaborn in this workflow. MethylFracAnalyzer processes bigwig files from SnakemakeMethylFrac for downstream analysis. It calculates percentiles for each feature, methylation rates from median methylated fractions and relative methylation rates. It computes the average methylated fraction in 100-kb windows (T2T v1.1 assembly) and methylation rates using average methylated fractions. It calculates the average methylated fraction relative to the TSS of active and inactive genes, and relative to CTCF sites, smoothed in 21-bp windows. Finally, it generates the associated figures. This software uses pandas, pyBigWig, matplotlib, seaborn and statsmodels (the original references are cited).

Data analysis

The code used to analyse the data is available at FigShare and Github:
Snakemake workflow to process the Illumina reads for methylated fraction analysis:
https://figshare.com/s/ebd9bc05ea5ff9734d2c?file=52109621
https://github.com/zhuweix/snakemakeMethylFrac
Downstream analysis of methylated fraction from the snakemake pipeline:
https://figshare.com/s/a57c2d50e912c14c94ba?file=53288837
https://github.com/zhuweix/methylFracAnalyzer

For manuscripts utilizing custom algorithms or software that are central to the research but not yet described in published literature, software must be made available to editors and reviewers. We strongly encourage code deposition in a community repository (e.g. GitHub). See the Nature Portfolio guidelines for submitting code & software for further information.

Data

Policy information about availability of data

All manuscripts must include a data availability statement. This statement should provide the following information, where applicable:

- Accession codes, unique identifiers, or web links for publicly available datasets
- A description of any restrictions on data availability
- For clinical datasets or third party data, please ensure that the statement adheres to our policy

The Illumina sequence data generated in this study have been deposited in the GEO database under the following accession codes:
GSE282872 [https://www.ncbi.nlm.nih.gov/geo/query/acc.cgi?acc=GSE282872] Methylation data for MCF10A cells.
GSE282873 [https://www.ncbi.nlm.nih.gov/geo/query/acc.cgi?acc=GSE282873] Methylation data for MCF7 cells.
GSE282874 [https://www.ncbi.nlm.nih.gov/geo/query/acc.cgi?acc=GSE282874] MNase-seq data for MCF7 cells before adenovirus treatment.
GSE292647 [https://www.ncbi.nlm.nih.gov/geo/query/acc.cgi?acc=GSE292647] RNA-seq data for MCF7 cells.
GSE292648 [https://www.ncbi.nlm.nih.gov/geo/query/acc.cgi?acc=GSE292648] Methylation data for MCF7 nuclei (no spermidine).
GSE292649 [https://www.ncbi.nlm.nih.gov/geo/query/acc.cgi?acc=GSE292649] MNase-seq data for MCF7 cells after adenovirus treatment.
Previously published data used in this study are available at the GEO database under the following accession codes:
GSE201262 [https://www.ncbi.nlm.nih.gov/geo/query/acc.cgi?acc=GSE201262] MCF7 ATAC-seq and RNA-seq data.
GSE237066 [https://www.ncbi.nlm.nih.gov/geo/query/acc.cgi?acc=GSE237066] MCF10A RNA-seq data.
GSE152410 [https://www.ncbi.nlm.nih.gov/geo/query/acc.cgi?acc=GSE152410] MCF10A ATAC-seq data.
GSE85158 [https://www.ncbi.nlm.nih.gov/geo/query/acc.cgi?acc=GSE85158] MCF7 and MCF10A ChIP-seq data.

Research involving human participants, their data, or biological material

Policy information about studies with human participants or human data. See also policy information about sex, gender (identity/presentation), and sexual orientation and race, ethnicity and racism.

Reporting on sex and gender	N/A
Reporting on race, ethnicity, or other socially relevant groupings	N/A
Population characteristics	N/A
Recruitment	N/A
Ethics oversight	N/A

Note that full information on the approval of the study protocol must also be provided in the manuscript.

Field-specific reporting

Please select the one below that is the best fit for your research. If you are not sure, read the appropriate sections before making your selection.

[x] Life sciences [ ] Behavioural & social sciences [ ] Ecological, evolutionary & environmental sciences

For a reference copy of the document with all sections, see nature.com/documents/nr-reporting-summary-flat.pdf

Life sciences study design

All studies must disclose on these points even when the disclosure is negative.

Sample size

All of our experiments involved millions of cells.

Data exclusions	Data for GATC sites with neighbouring GATC sites closer than 150 bp on both sides were excluded due to differential loss of short DNA fragments during library preparation. Also GATC sites overlapping CG were excluded. Described in detail in Methods.
Replication	Two independent biological replicate experiments were performed for each genomic sample. Parallel analysis of replicate experiments is provided in Extended Data. All replicate experiments were successful.
Randomization	N/A
Blinding	Blinding is not performed in this type of study (cell line genomics).

Reporting for specific materials, systems and methods

We require information from authors about some types of materials, experimental systems and methods used in many studies. Here, indicate whether each material, system or method listed is relevant to your study. If you are not sure if a list item applies to your research, read the appropriate section before selecting a response.

Materials & experimental systems

n/a	Involved in the study
☐	☒ Antibodies
☐	☒ Eukaryotic cell lines
☒	☐ Palaeontology and archaeology
☒	☐ Animals and other organisms
☒	☐ Clinical data
☒	☐ Dual use research of concern
☒	☐ Plants

Methods

n/a	Involved in the study
☒	☐ ChIP-seq
☐	☒ Flow cytometry
☒	☐ MRI-based neuroimaging

Antibodies

Antibodies used	HRP-conjugated anti-HA antibody 3F10 (Roche 12013819001) used at 1:5,000 dilution in 5% skim milk in TBS (20mM Tris-HCl pH 8.0, 0.5 M NaCl) with 0.1% Tween-20 overnight at 4oC. HRP-conjugated anti-tubulin antibody (Abcam ab-185067; lot 1058457-2) diluted 1:20,000 in PBS/0.1% Tween-20 with 0.5% skim milk for 1 h at room temperature with rotation.
Validation	The anti-HA antibody is raised against a haemagglutinin (HA) epitope used for protein tagging. It has been validated by the manufacturer (https://www.sigmaaldrich.com/US/en/product/roche/12013819001#product-documentation). We have observed that this antibody does not give a signal with MCF7 and MCF10A cell extracts before adenovirus transduction. The anti-tubulin antibody was used as a loading control for Western blots. This antibody was validated by the manufacturer for mammalian tubulins (https://www.abcam.com/products/primary-antibodies/hrp-alpha-tubulin-antibody-epr13478b-loading-control-ab185067.html).

Eukaryotic cell lines

Policy information about cell lines and Sex and Gender in Research

Cell line source(s)	MCF7 (ATCC HTB-22) and MCF10A (ATCC CRL-10317) are human female breast cell lines.
Authentication	MCF10A cells were obtained directly from ATCC with their authentication guarantee. MCF7 cells were sent to ATCC for authentication. They did the following: Seventeen short tandem repeat (STR) loci plus the gender determining locus, Amelogenin, were amplified using the commercially available PowerPlex® 18D Kit from Promega. The cell line sample was processed using the ABI Prism® 3500xl Genetic Analyzer. Data were analyzed using GeneMapper® ID-X software (Applied Biosystems). Appropriate positive and negative controls were run and confirmed for each sample submitted..
Mycoplasma contamination	Not tested by us.
Commonly misidentified lines (See ICLAC register)	No commonly misidentified cell lines were used in this study.

Plants

Seed stocks	N/A
Novel plant genotypes	N/A
Authentication	N/A

Flow Cytometry

Plots

Confirm that:

- [ ] The axis labels state the marker and fluorochrome used (e.g. CD4-FITC).
- [ ] The axis scales are clearly visible. Include numbers along axes only for bottom left plot of group (a 'group' is an analysis of identical markers).
- [ ] All plots are contour plots with outliers or pseudocolor plots.
- [ ] A numerical value for number of cells or percentage (with statistics) is provided.

Methodology

Sample preparation	Propidium iodide staining and flow cytometric DNA analysis of MCF7 and MCF10A cells were performed as follows 47. Cells (0.1-0.2 million) were resuspended in 50 µl cold buffer (250 mM sucrose, 40 mM trisodium citrate, 5% v/v DMSO) and frozen at -80°C. For FACS, cells were thawed and 200 µl of ice-cold Solution A (0.03 mg/ml trypsin, 3.4 mM trisodium citrate, 0.1% v/v NP-40, 1.5 mM spermine tetrahydrochloride, 0.5 mM Tris-HCl pH 7.6) was added. The mixture was incubated at room temperature for 5 min. Subsequently, 100 µl of ice-cold Solution B (0.5 mg/ml trypsin inhibitor, 0.1 mg/ml RNase A, 3.4 mM trisodium citrate, 0.1% v/v NP-40, 1.5 mM spermine tetrahydrochloride, 0.5 mM Tris-HCl pH 7.6) was added and incubated for another 5 min at room temperature. Finally, 20 µl propidium iodide at 1 mg/ml (Invitrogen P3566) was added and incubated at room temperature in the dark to prevent photobleaching. The cells were analysed using a FACSCalibur flow cytometer (Becton Dickinson) and Cell Quest Pro software, following the manufacturer's instructions.
Instrument	FACSCalibur flow cytometer (Becton Dickinson)
Software	Cell Quest Pro software
Cell population abundance	FACS was used only to confirm that confluent cells were arrested in G1 (see Supplementary Fig. 6c).
Gating strategy	No sorting was done.

- [ ] Tick this box to confirm that a figure exemplifying the gating strategy is provided in the Supplementary Information.
