# Supplementary material for: Nucleosome dynamics render heterochromatin accessible in living human cells
Source: Nat Commun. 2025 May 16;16:4577. doi: 10.1038/s41467-025-59994-7 (PMC12084565; doi:10.1038/s41467-025-59994-7)
Supplement: Supplementary file 4 — Source Data [file 41467_2025_59994_MOESM4_ESM.zip › 250422_Source_Data.pdf]

Source Data

Nucleosome dynamics render heterochromatin accessible in living human cells

Hemant K. Prajapati, Zhuwei Xu, Peter R. Eriksson and David J. Clark

Division of Developmental Biology, *Eunice Kennedy-Shriver* National Institute of Child Health and Human Development, National Institutes of Health, Bethesda MD 20892, USA.

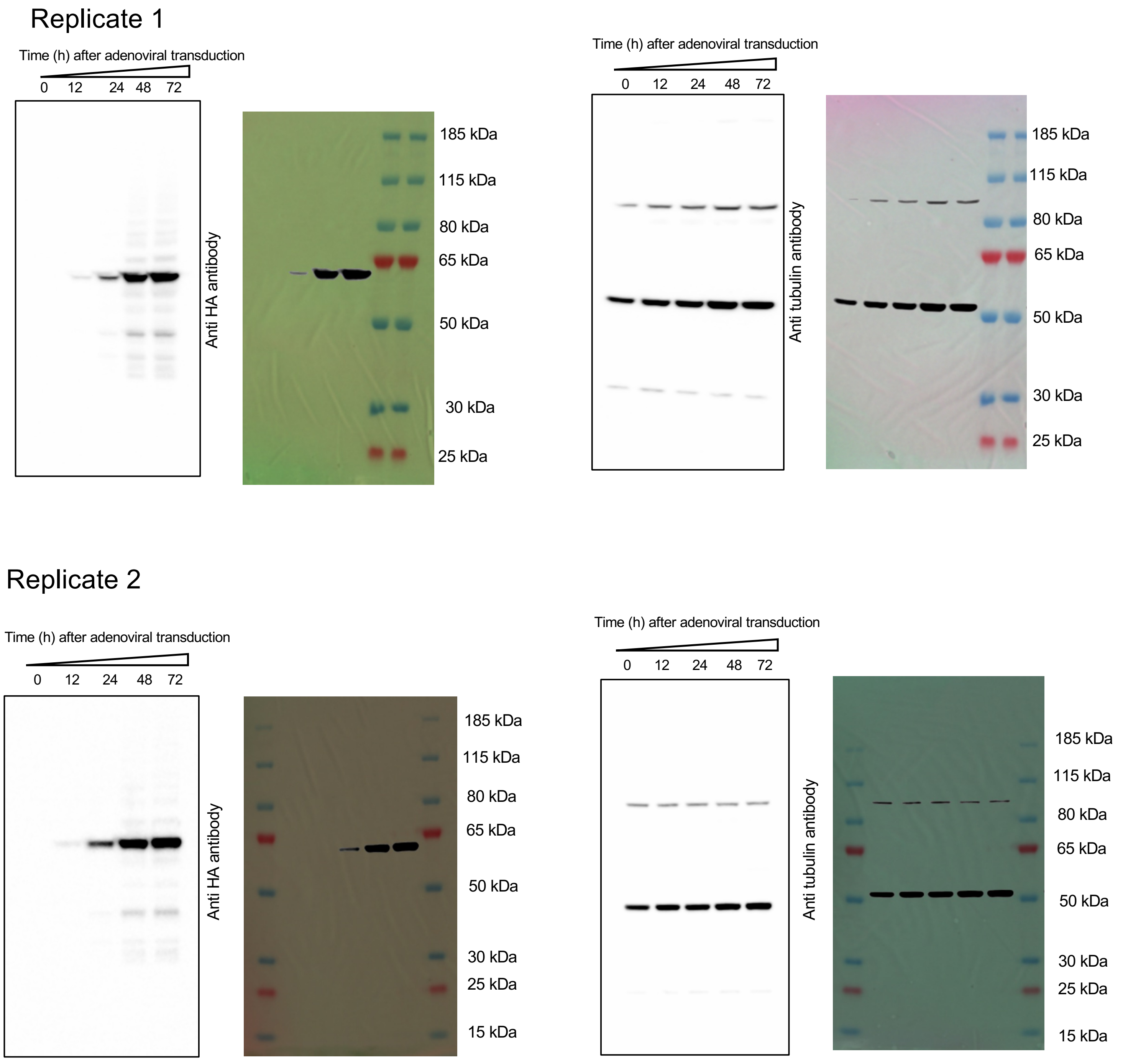
Replicate 1
Time (h) after adenoviral transduction
0 12 24 48 72
Anti HA antibody
185 kDa
115 kDa
80 kDa
65 kDa
50 kDa
30 kDa
25 kDa
Time (h) after adenoviral transduction
0 12 24 48 72
Anti tubulin antibody
185 kDa
115 kDa
80 kDa
65 kDa
50 kDa
30 kDa
25 kDa
Replicate 2
Time (h) after adenoviral transduction
0 12 24 48 72
Anti HA antibody
185 kDa
115 kDa
80 kDa
65 kDa
50 kDa
30 kDa
25 kDa
15 kDa
Time (h) after adenoviral transduction
0 12 24 48 72
Anti tubulin antibody
185 kDa
115 kDa
80 kDa
65 kDa
50 kDa
30 kDa
25 kDa
15 kDa

Fig. 1b

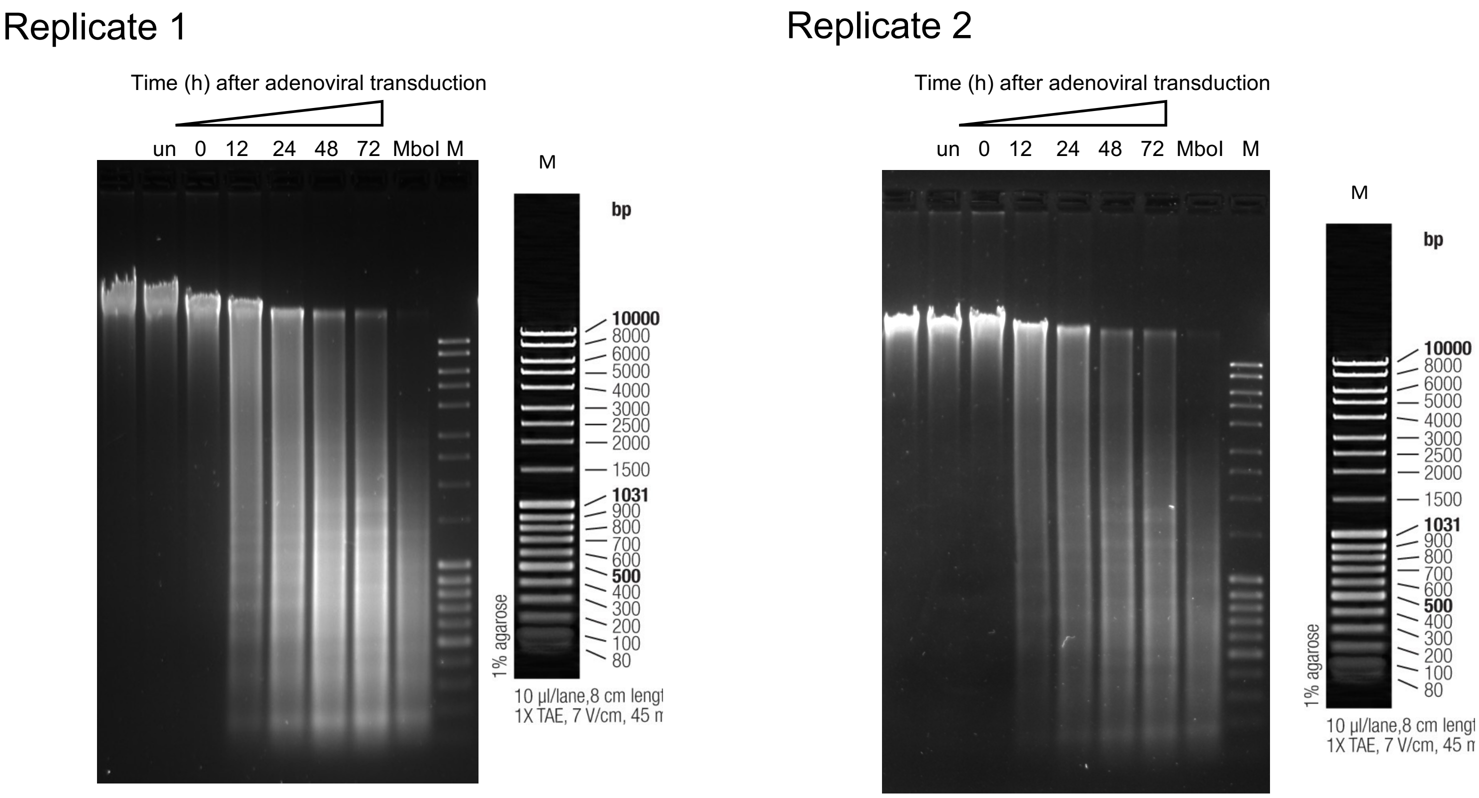
Replicate 1
Time (h) after adenoviral transduction
un 0 12 24 48 72 MboI M
M
bp
10000
8000
6000
5000
4000
3000
2500
2000
1500
1031
900
800
700
600
500
400
300
200
100
80
1% agarose
10 µl/lane,8 cm lengt
1X TAE, 7 V/cm, 45 m
Replicate 2
Time (h) after adenoviral transduction
un 0 12 24 48 72 MboI M
M
bp
10000
8000
6000
5000
4000
3000
2500
2000
1500
1031
900
800
700
600
500
400
300
200
100
80
1% agarose
10 µl/lane,8 cm lengt
1X TAE, 7 V/cm, 45 m

Fig. 1c

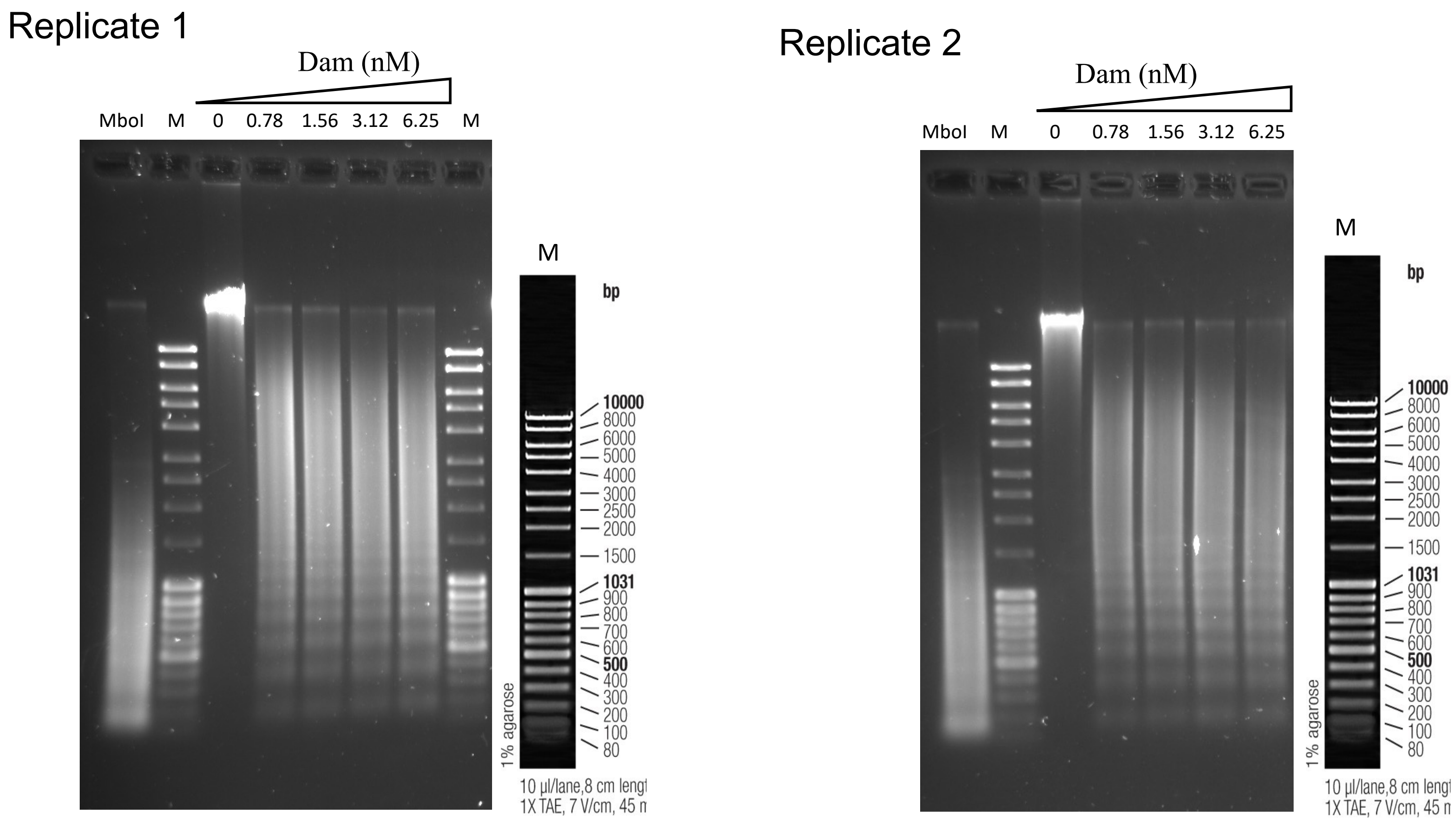

Replicate 1
Dam (nM)
MboI M 0 0.78 1.56 3.12 6.25 M
M
bp
10000
8000
6000
5000
4000
3000
2500
2000
1500
1031
900
800
700
600
500
400
300
200
100
80
1% agarose
Replicate 2
Dam (nM)
MboI M 0 0.78 1.56 3.12 6.25
M
bp
10000
8000
6000
5000
4000
3000
2500
2000
1500
1031
900
800
700
600
500
400
300
200
100
80
1% agarose

Fig. 4a

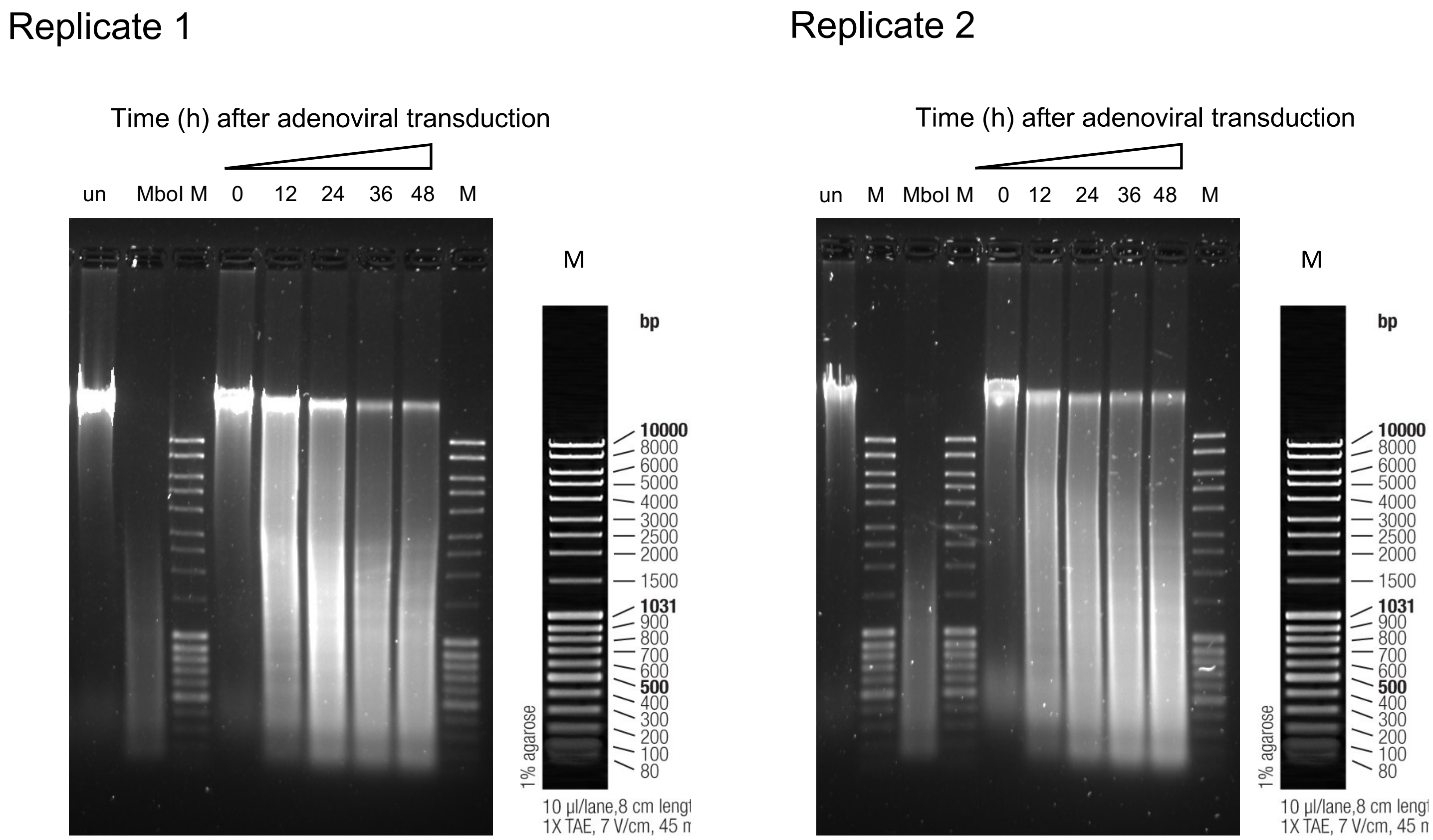

Supplementary Fig. 5a

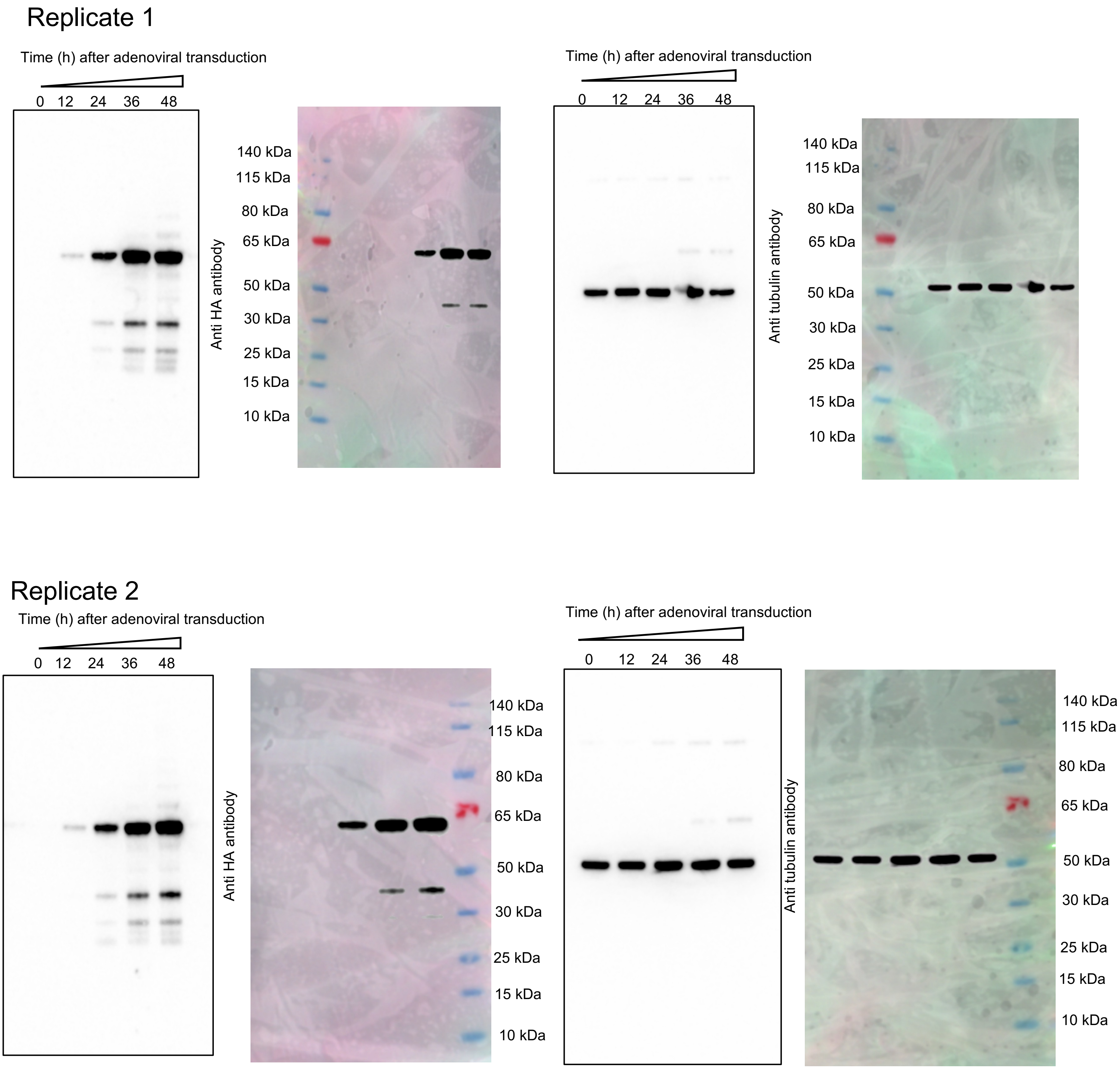

Supplementary Fig. 5b

Replicate 1

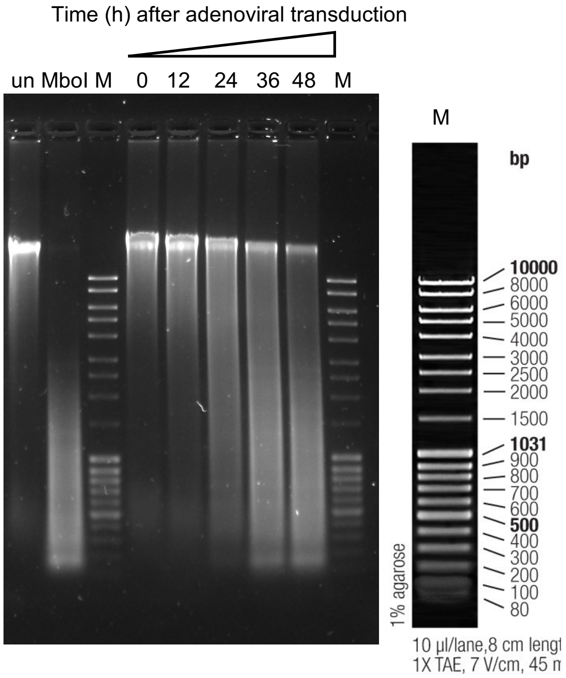

Replicate 2

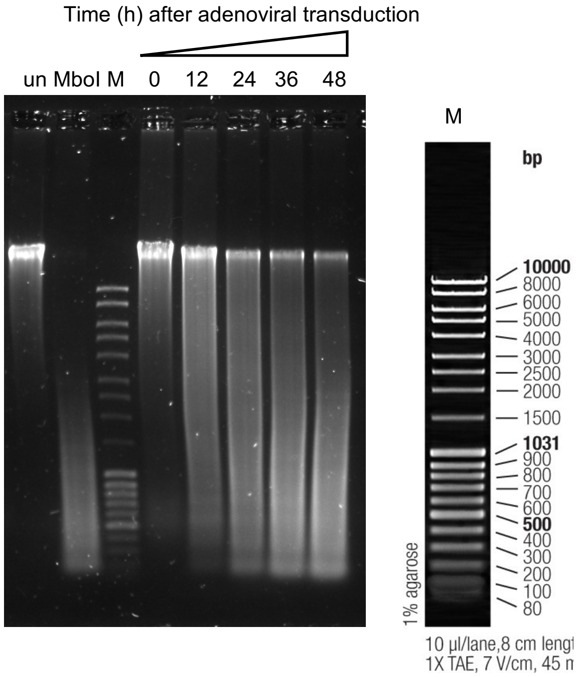

Supplementary Fig. 6a

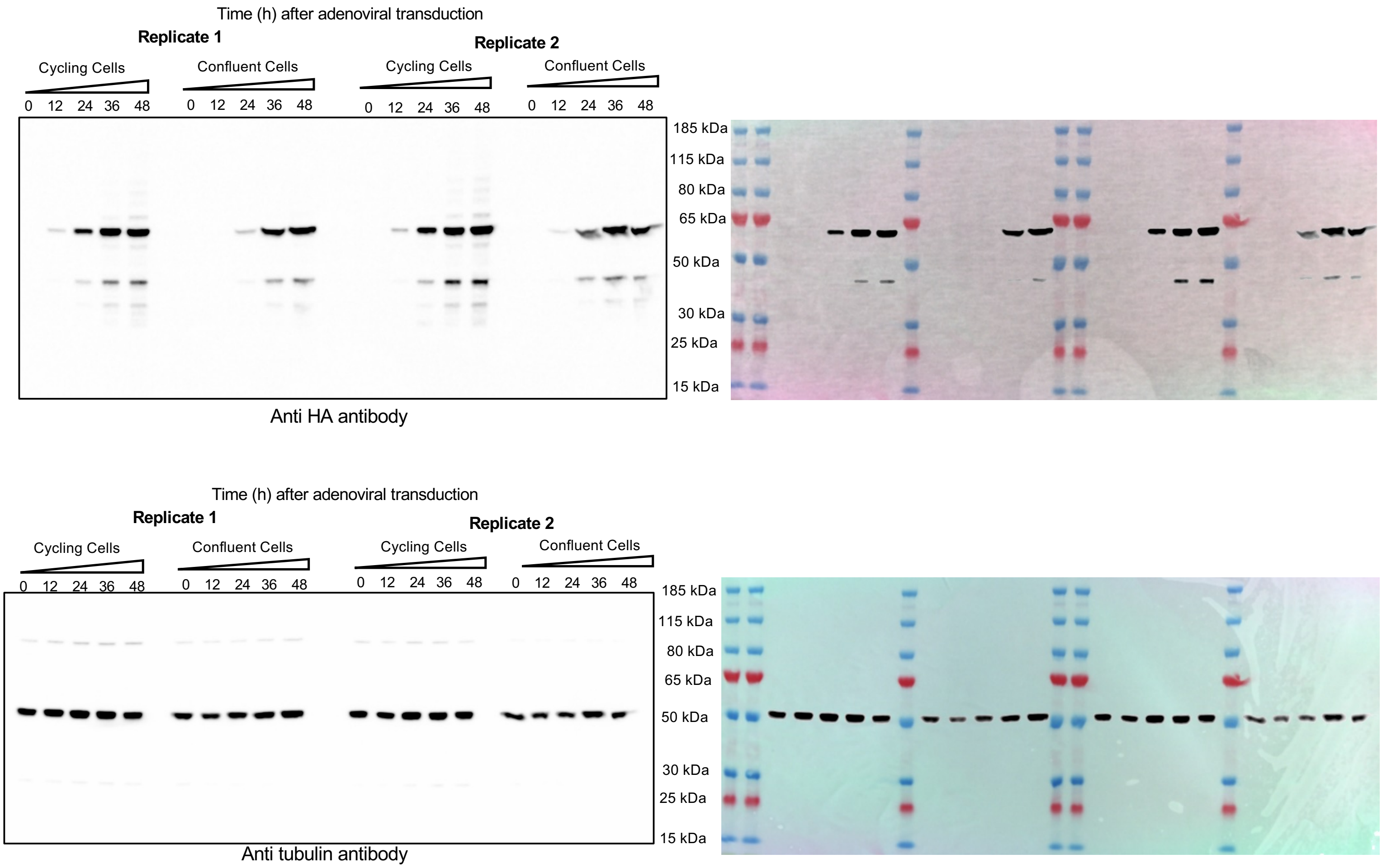

Supplementary Fig. 6b
